# NMB promotes the progression of colorectal cancer by regulating the NF-κB/P65 signaling pathway

**DOI:** 10.3389/fimmu.2025.1596451

**Published:** 2025-05-23

**Authors:** Jiaxin Fan, Yuming Liu, Jiajia Wang, Tian Yao, Shaoxiong Bai, Xiaole Ma, Hao Chen, Yuhao Chen, Huiyang Gao, Yuntong Guo, He Huang

**Affiliations:** ^1^ Department of Gastrointestinal Surgery, First Hospital of Shanxi Medical University, Taiyuan, Shanxi, China; ^2^ First Clinical Medical College, Shandong University, Jinan, China; ^3^ Department of Geriatrics, Shanxi Provincial People’s Hospital, Taiyuan, China

**Keywords:** colorectal cancer, NMB, biomarker, deubiquitination, prognosis

## Abstract

**Background:**

Colorectal carcinoma (CRC) ranks as the fourth most prevalent malignancy globally. Neuromedin B (NMB) and its corresponding receptor have been implicated in the pathogenesis of multiple neoplastic conditions. Nevertheless, the specific involvement of NMB in CRC progression remains poorly characterized.

**Methods:**

To investigate the differential expression of NMB, data were extracted from the TCGA and GEO databases, supplemented by tissue microarrays derived from clinical specimens. Prognostic significance was assessed through Kaplan-Meier survival analysis, ROC curve evaluation, Cox proportional hazards regression, clinical correlation studies, and nomogram construction. Experimental validation of NMB expression in CRC was performed using qRT-PCR, western blotting, immunohistochemistry, and ELISA techniques. Functional characterization of NMB's tumor-regulatory capacity was conducted through colony formation assays, MTT proliferation tests, wound healing experiments, and transwell migration/invasion assays. Mechanistic insights were obtained by identifying upstream regulatory proteins via co-immunoprecipitation and exploring potential signaling pathways through gene set enrichment analysis (GSEA) combined with western blot validation.

**Results:**

Analysis of clinical data revealed that NMB exhibits markedly elevated expression levels in CRC tissues, with higher expression correlating significantly with poor prognosis. Receiver operating characteristic curve analysis validated NMB's utility as a reliable prognostic biomarker for survival outcomes. Importantly, multivariate Cox regression analysis demonstrated NMB’s independent prognostic value in CRC patients. *In vitro* functional studies provided compelling evidence that NMB promotes CRC cell proliferation, migration, and invasive potential. Molecular investigations uncovered that NMB protein stability is regulated through USP21-dependent deubiquitination, leading to subsequent activation of the NF-κB signaling cascade and tumor progression. Notably, NMB expression patterns showed significant correlation with 5-fluorouracil resistance profiles, indicating its potential role in mediating chemotherapeutic response in CRC treatment regimens.

**Conclusion:**

NMB exhibits significant overexpression in colorectal cancer, demonstrating promising potential as a dual-functional biomarker for both diagnostic identification and prognostic evaluation in CRC cases.

## Introduction

Cancer significantly impacts morbidity and mortality rates in both developed and developing countries (1). Limited comprehension of cancer’s pathological processes and regulatory pathways suggests a projected increase in mortality rates (2). While advancements in therapeutic interventions have enhanced prognosis for certain malignancies, treatment outcomes remain suboptimal for many cancer types (3). Colorectal cancer (CRC) represents a particularly significant global health challenge, ranking as the fourth most prevalent malignancy worldwide and accounting for approximately 90,000 annual deaths (4). Current therapeutic approaches, despite their diversity, have failed to substantially improve the 5-year survival rate for CRC patients (5, 6). This underscores the critical need for identifying novel biomarkers and elucidating the underlying molecular mechanisms of CRC to develop more effective therapeutic strategies.

NMB (Neuromedin B), a protein-coding gene belonging to the bombesin-like neuropeptide family, plays a crucial role in appetite regulation through its anorexigenic effects. This gene product mediates colonic smooth muscle contraction via interaction with its cognate receptor (NMBR), which exhibits extensive expression throughout the gastrointestinal system (7). Emerging evidence has established significant associations between NMB expression patterns and multiple oncogenic processes, including tumorigenesis, progression, recurrence, and metastatic dissemination in various malignancies such as breast cancer (8) and lung cancer (9). Notably, immunohistochemical analyses have revealed co-localization of NMB and its receptors in proliferating colonic epithelial cells, suggesting autocrine signaling mechanisms in both normal and neoplastic conditions (10). Despite these findings, the precise molecular mechanisms underlying NMB’s involvement in colorectal carcinogenesis remain poorly characterized, warranting further investigation.

Through comprehensive analysis, this study elucidated the functional significance and expression dynamics of NMB in colorectal carcinogenesis, revealing marked upregulation in neoplastic tissues compared to normal counterparts. Clinical correlation analysis demonstrated that heightened NMB expression levels were strongly associated with advanced disease characteristics and unfavorable clinical outcomes. We developed a novel prognostic model incorporating NMB expression metrics and validated its predictive value for overall survival rates. *In vitro* functional studies demonstrated that NMB overexpression significantly augmented malignant phenotypes, including cellular proliferation, migratory capacity, and invasive potential in CRC cell lines. Mechanistic investigations identified USP21-mediated deubiquitination as the principal regulatory mechanism governing NMB protein stabilization, which subsequently triggers NF-κB pathway activation. Furthermore, chemosensitivity profiling suggested that NMB expression status may influence 5-fluorouracil (5-FU) responsiveness, potentially affecting treatment outcomes. These collective findings establish NMB as a critical molecular driver in CRC pathogenesis and support its dual utility as a prognostic indicator and potential therapeutic target.

## Materials and methods

### Data processing

The study utilized comprehensive genomic data obtained from two principal repositories. From The Cancer Genome Atlas (TCGA) accessible via https://portal.gdc.cancer.gov/repository), we acquired microarray datasets comprising 612 samples (44 normal and 568 tumor specimens) alongside clinical survival data for 711 colorectal cancer patients. Furthermore, NMB expression profiles and corresponding clinical metadata were retrieved from datasets (GSE9348, GSE20482, GSE21510, GSE23878, GSE32323, GSE17536, GSE17538, GSE29623, GSE38832, GSE40967, GSE71187, GSE87211, GSE103479) through the Gene Expression Omnibus (GEO; https://www.ncbi.nlm.nih.gov/geo/) platform. To ensure data integrity, we implemented a Perl-based computational pipeline for the systematic integration of gene expression profiles with clinical annotations, which simultaneously filtered out records with missing or ambiguous clinical information.

### Differential analysis of gene expression

Statistical analyses were performed using specialized R packages (version 4.4.2) to evaluate NMB expression patterns in CRC. Differential expression analysis between malignant and normal tissues was conducted through the limma package, while survival analysis and data visualization were accomplished using the Survival and beeswarm packages, respectively. Graphical representations of expression differences were generated through boxplots, incorporating both scatter and paired difference plots to ensure comprehensive data interpretation. To validate NMB protein expression in CRC tissues, we queried the Human Protein Atlas database (HPA; http://www.proteinatlas.org/), a comprehensive repository of protein expression data across human tissues and cancers.

### Survival analysis

The study cohort was dichotomized into distinct subgroups according to median NMB expression thresholds: low-expression and high-expression categories. Survival outcomes were evaluated through Kaplan-Meier analysis, implemented using the survival and survminer packages, with corresponding K-M curves generated for visual interpretation. To enhance statistical robustness, we performed a meta-analysis integrating data from both TCGA and GEO repositories, systematically evaluating the prognostic significance of NMB expression in colorectal cancer. Furthermore, predictive accuracy was quantitatively assessed through ROC curve analysis, utilizing the timeROC package to determine NMB’s prognostic efficacy in CRC patient survival.

### Clinical correlation analysis

To investigate potential relationships between NMB expression and clinical features, comprehensive statistical analyses were conducted. Demographic factors (age and sex) and metastatic status were evaluated using the Wilcoxon rank-sum test, a non-parametric method suitable for comparing independent samples. Tumor-specific characteristics, including T stage, N stage, and overall staging, were analyzed through the Kruskal-Wallis test, which is particularly appropriate for assessing multiple categorical variables. The expression patterns of NMB across these clinical parameters were subsequently visualized using boxplot representations, enabling clear identification of statistically significant intergroup variations.

### Cox regression analyses

To evaluate prognostic factors in colorectal cancer, we employed Cox proportional hazards regression models in both univariate and multivariate analyses. The initial univariate analysis examined the predictive value of individual clinicopathological variables and NMB expression levels on patient survival outcomes. Building upon these results, a multivariate Cox regression model was implemented to ascertain the independent prognostic significance of NMB expression after adjusting for other relevant clinical factors. Statistical computations were executed using the survival and Survminer packages, with the results graphically presented in a comprehensive forest plot to illustrate hazard ratios and their corresponding confidence intervals.

### Gene set enrichment analysis

To elucidate the molecular mechanisms underlying NMB’s role in colorectal cancer, we performed Gene Set Enrichment Analysis (GSEA, version 4.1.0) to identify associated signaling pathways. The analysis was stratified according to NMB expression phenotypes, utilizing the curated Kyoto Encyclopedia of Genes and Genomes (KEGG) pathway database (c2.cp.kegg.v7.2.symbols.gmt) as the reference gene set. Pathway enrichment was determined through 1000 permutations of gene sets, generating three key statistical parameters: normalized enrichment score (NES), nominal p-value, and false discovery rate (FDR) q-value, which collectively enabled rigorous ranking of significantly enriched pathways. This systematic approach revealed multiple oncogenic pathways that were significantly associated with NMB expression patterns in CRC.

### Immune infiltration analyses

To elucidate NMB’s immunoregulatory functions in colorectal cancer development, we systematically examined its association with tumor immune microenvironment characteristics. The analytical workflow commenced with immune cell quantification through computational pipelines integrating E1071, preprocessCore, and Limma packages. Subsequent visualization of immune infiltration patterns was achieved via histogram generation using the Corrplot package. Patient stratification into high and low NMB expression groups facilitated comparative immune profiling, with differential analyses executed through Limma and Vioplot packages (significance level: P<0.05). Bivariate correlation assessments between NMB expression and immune cell composition were subsequently performed, leveraging the combined functionality of Limma, ggplot2, ggpubr, and ggExtra packages (P<0.05). The convergent findings from differential and correlation analyses identified distinct immune cell subsets exhibiting significant associations with NMB expression profiles. Complementing these investigations, we employed the somatic copy number alterations (SCNA) module to assess potential linkages between NMB genomic variations and six principal leukocyte subpopulations. Through this integrated analytical framework, we uncovered novel mechanistic insights into NMB-mediated regulation of immune microenvironment dynamics during colorectal cancer pathogenesis.

### Analysis of gene markers of tumor invasive immune cells

We employed the Tumor Immune Estimation Resource (TIMER) platform to systematically analyze immune cell infiltration patterns across 32 distinct cancer types, utilizing a dataset of 10,897 samples derived from The Cancer Genome Atlas (TCGA). Through the platform’s correlation module, we examined associations between NMB expression profiles and specific gene markers of tumor-infiltrating immune cells. This investigation sought to elucidate potential interactions between NMB expression dynamics and immune cell infiltration characteristics, thereby contributing to our understanding of its immunomodulatory functions within diverse tumor microenvironments.

### Construction of the prognostic signature

In this investigation, we employed the rms package in R to integrate survival data with seven clinical parameters: NMB expression, age, sex, T stage, N stage, M stage, and overall stage. Using Cox regression analysis on a cohort of 472 samples, we developed a nomogram to evaluate the prognostic value of these variables. The predictive model assigned individual risk scores based on regression coefficients (coef) derived from multivariate Cox regression analysis, with the formula: risk score = coefNMB×NMB expression + coefage×age + coefsex×gender + coefT×T + coefN×N + coefM×M + coefstage×stage. These coefficients were calculated from the logarithmic transformation of hazard ratios (HR). Patients were subsequently divided into high-risk and low-risk categories using the median risk score as the threshold, enabling prognostic stratification and therapeutic decision-making.

Survival analysis was conducted using the Kaplan-Meier method to compare overall survival (OS) between risk groups, with results visualized through survival curves. To assess the predictive accuracy of the risk score and clinical features in colorectal cancer (CRC), we generated receiver operating characteristic (ROC) curves using the survivalROC package. The area under the curve (AUC) values were computed to determine the sensitivity and specificity of these prognostic indicators.

### Drug sensitivity analysis

We employed the Linked Omics platform to systematically identify genes exhibiting co-expression patterns with NMB. Subsequently, we assessed the pharmacological sensitivity profiles of these NMB-associated genes through Gene Set Cancer Analysis (GSCA), utilizing the publicly accessible web resource (http://bioinfo.life.hust.edu.cn/web/GSCALite/).

### Cell culture

The human colorectal epithelial cell line NCM460 and six CRC cell lines (HCT116, DLD1, SW480, SW620, Caco2, and HT29) were procured from Wuhan Punosai Life Technology Co., Ltd. All cell lines were maintained in Dulbecco’s Modified Eagle Medium (DMEM) containing 10% fetal bovine serum (FBS) under standard culture conditions (37°C, 5% CO2, humidified atmosphere). For gene knockdown experiments, cells seeded in 6-well plates were transfected with NMB-specific shRNA or control vectors (WeiZhen, Shandong, China) using Lipofectamine 3000 transfection reagent (Invitrogen, USA). Post-transfection analyses were performed after 48 hours, with all experimental conditions replicated in triplicate to ensure data reliability.

### Quantitative reverse transcription polymerase chain reaction

Total RNA was isolated from cells using TRIzol reagent (Invitrogen, USA), followed by quantitative reverse transcription polymerase chain reaction (qRT-PCR) analysis performed according to established protocols. Gene-specific primers targeting NMB were commercially obtained from BioSune (Shanghai, China).

### Clinical samples

A retrospective cohort study was performed at the Department of Gastrointestinal Surgery, First Hospital of Shanxi Medical University, encompassing 61 CRC patients treated between January 2019 and December 2021. The inclusion criteria comprised: (1) histopathologically verified CRC diagnosis, (2) absence of prior anticancer therapy, (3) accessibility of paraffin-embedded tissue specimens for immunohistochemical evaluation, and (4) comprehensive medical documentation. Exclusion criteria included: (1) presence of synchronous malignancies or metastatic disease, and (2) incomplete follow-up data. Patient outcomes were monitored through systematic review of medical records and structured telephone interviews. OS served as the primary study endpoint, with the observation period concluding on July 4, 2024. The Institutional Review Board (IRB) of the First Hospital of Shanxi Medical University (Approval No. KYYJ-2025-131) granted an exemption for informed consent requirements.

### Western blot

Protein extraction was performed by lysing cells and tissues on ice, followed by quantification of protein concentration using the BCA assay. Protein samples were mixed with loading buffer, heat-denatured at 100°C for 10 minutes, and subsequently stored at -20°C for further analysis. For immunoblotting, 20 μg of total protein was separated by SDS-PAGE and transferred onto PVDF membranes. Membranes were blocked with 5% non-fat milk in TBST and probed with primary antibodies at 4°C overnight. Following primary antibody incubation, membranes were treated with horseradish peroxidase-conjugated secondary antibodies at room temperature for 1 hour. Protein bands were visualized using enhanced chemiluminescence detection and imaged with a gel documentation system. GAPDH served as the loading control throughout the experiments. The following primary antibodies were utilized: anti-NMB (Proteintech, 10888-1-AP), anti-USP21 (Santa Cruz Biotechnology, sc-515911), anti-NF-κBp65/RelA (ABclonal, A2547), anti-phospho-NF-κBp65/RelA-S536 (ABclonal, AP0475), anti- RAF (Proteintech, 66592-1-lg), and anti-RAS (Proteintech, 81615-1-RR).

### Co-immunoprecipitation assay

Following complete resuspension of magnetic beads, 30 μL of Protein A/G magnetic beads were aliquoted and subjected to two washes with 400 μL of binding/washing buffer. Magnetic beads were subsequently conjugated with USP21 antibodies at concentrations ranging from 5 to 50 μg/mL, followed by incubation at ambient temperature for 60 minutes. Post-incubation, magnetic separation was performed, and the beads were washed four times with binding/washing buffer. Cell lysates were then introduced to the antibody-conjugated magnetic beads and incubated at room temperature for 1 hour with continuous agitation using a flip mixer. After magnetic separation, the beads underwent four additional washing cycles. Following supernatant removal, 30 μL of 1× SDS-PAGE loading buffer was added to the magnetic beads, thoroughly mixed, and heated to 95°C for 5 minutes. The magnetic beads were subsequently separated, and the resulting supernatant was collected for subsequent SDS-PAGE analysis.

### Deubiquitination assay

To initiate the experiment, cells were exposed to the proteasome inhibitor MG132 at a concentration of 10 μM for 6 hours prior to harvesting. Cellular proteins were extracted using RIPA lysis buffer supplemented with ultrasonic disruption. Following protein denaturation for 10 minutes, immunoprecipitation was performed using anti-NMB antibody. Subsequently, ubiquitination levels were quantitatively assessed through western blot analysis.

### Immunohistochemistry

Paraffin-embedded tissue specimens from 61 CRC patients were processed to construct tissue microarray blocks. Immunohistochemical analysis was performed according to established protocols. Tissue sections underwent antigen retrieval by heating in citric acid buffer (pH 6.0) for 15 minutes. Endogenous peroxidase activity was neutralized through treatment with 3% methanol-hydrogen peroxide solution. Primary antibodies targeting NMB and Ki67 (Abmart, TW0001) were applied and incubated at 4°C for 12 hours. Following thorough washing, sections were incubated with biotinylated secondary antibodies for 50 minutes at room temperature. Chromogenic development was achieved using 3,3’-diaminobenzidine (DAB), followed by hematoxylin counterstaining and permanent mounting. Microscopic evaluation was conducted using a light microscope. NMB expression levels were quantitatively evaluated by two independent, blind pathologists without access to clinical data.

### Enzyme-linked immunosorbent assay

The quantification of NMB cytokine levels in both human tissue homogenates and serum samples was performed using a high-sensitivity ELISA kit (R&D Systems, Inc., Minneapolis, MN, USA; catalog number ELM-NMB-1). All experimental procedures were strictly conducted in accordance with the manufacturer’s established protocol.

### MTT cell proliferation assay

To assess cell proliferation, HCT116 cells transfected with either NMB-targeting shRNA or empty vector were plated in 96-well plates at a density of 5×10³ cells per well. Peripheral wells were supplemented with sterile PBS to minimize edge effects, with experimental replicates distributed across four independent plates. Cells were maintained under standard culture conditions (37°C, 5% CO2) for subsequent analysis.

Following 24-hour incubation, cellular metabolic activity was evaluated using the MTT assay. Each well received 20 µL of MTT solution (5 mg/mL, 0.5% w/v) and was incubated for 4 hours to allow formazan crystal formation. The culture medium was then carefully aspirated, and intracellular formazan was solubilized with 150 µL DMSO through gentle agitation (10 minutes, low-speed orbital shaker).

Optical density (OD) measurements were performed at 450 nm using a multi-functional microplate reader. To monitor proliferation kinetics, MTT assays were conducted daily for four consecutive days, with OD values recorded at each time point. The acquired data were analyzed to generate growth curves, enabling quantitative comparison of cellular proliferation rates.

### Colony assay

To evaluate the impact of NMB knockdown on cellular growth, HCT116 cells were genetically modified with either NMB-specific shRNA or control vector. These cells were plated in 6-well culture dishes at a density of 1×10³ cells per well and maintained under standard culture conditions for 7 days. Following the incubation period, cell colonies were subjected to a standardized staining protocol. Colonies were first rinsed twice with phosphate-buffered saline (PBS) to remove residual media, then fixed using 4% paraformaldehyde solution. Cellular staining was performed with 0.1% crystal violet for 10 minutes at room temperature.

After the staining procedure, excess dye was removed through additional PBS washes, and the plates were air-dried at ambient temperature. Quantitative analysis was conducted by microscopic examination, where colony numbers were systematically counted to determine the effect of NMB suppression on HCT116 cell proliferation. This colony formation assay provided quantitative data on the clonogenic potential of NMB-knockdown cells compared to vector controls.

### Wound-healing assay

To evaluate cellular migration capabilities, a standardized Wound-Healing assay was performed using HCT116 cells with either NMB-targeting shRNA or control vector transfection. Prior to cell seeding, reference lines were drawn at the base of a 6-well plate using a black marker aligned with a ruler. Cells were plated at a density of 6×10^5^ cells per well and allowed to adhere for 24 hours under standard culture conditions. Following adhesion, a uniform scratch was created using a 10 μL pipette tip, with the starting point clearly marked for consistent measurement.

After scratch induction, wells were gently rinsed with phosphate-buffered saline (PBS) to remove non-adherent cells, followed by replenishment with 2 mL of low-serum medium (2% fetal bovine serum). The plates were then returned to the incubator for continued culture.

Cell migration was monitored at predetermined intervals (0-, 12-, and 24-hours post-scratch) using phase-contrast microscopy at 4× magnification. Digital images were captured for quantitative analysis, with wound closure areas measured using ImageJ software. This approach enabled precise quantification of migration rates, allowing for comparative assessment of NMB knockdown effects on cellular motility.

### Transwell assay

To assess cellular invasion and migration capabilities, a modified Transwell assay was conducted. Matrigel was initially diluted with serum-free medium at a 1:10 ratio, and 100 μL aliquots were carefully transferred to the upper chambers of Transwell inserts. The coated inserts were then incubated at 37°C for 4–6 hours to facilitate matrix solidification. Both experimental and control groups were prepared by adjusting cell concentrations to 1.5 × 10^5^ cells/mL in serum-free medium.

For the invasion assay, 200 μL of cell suspension was carefully loaded into each Matrigel-coated upper chamber, while the lower chambers received 500 μL of complete medium as a chemoattractant. Following a 24-hour incubation period, cells were fixed with 4% paraformaldehyde for 20 minutes at room temperature. After fixation, the inserts underwent two PBS washes before staining with 0.1% crystal violet solution for 20 minutes. Excess stain was removed through three PBS washes, and non-migrated cells were gently removed from the upper membrane surface using cotton swabs. In parallel migration experiments, the same protocol was followed without Matrigel coating to evaluate cell motility independent of matrix degradation.

Quantitative analysis was performed by capturing images from five randomly selected high-power fields per insert. Cell counts were determined using Adobe Photoshop’s image analysis tools, allowing for objective quantification of invasive and migratory potential.

### Statistical analysis

Statistical analyses were conducted using SPSS 26.0 (IBM Corp., Armonk, NY, USA) and R software (version 4.4.2; R Foundation for Statistical Computing, Vienna, Austria). Non-parametric tests, including the Wilcoxon signed-rank test and Kruskal-Wallis H test, were applied to examine the association between NMB expression levels and clinicopathological characteristics. Survival outcomes were evaluated through Kaplan-Meier (K-M) analysis, with log-rank tests used to compare differences in survival curves based on NMB expression levels. Prognostic factors were assessed using Cox proportional hazards regression models, incorporating both univariate and multivariate analyses to identify independent predictors of survival.

## Results

### Expression and prognosis of NMB in databases

**Figure 1:**
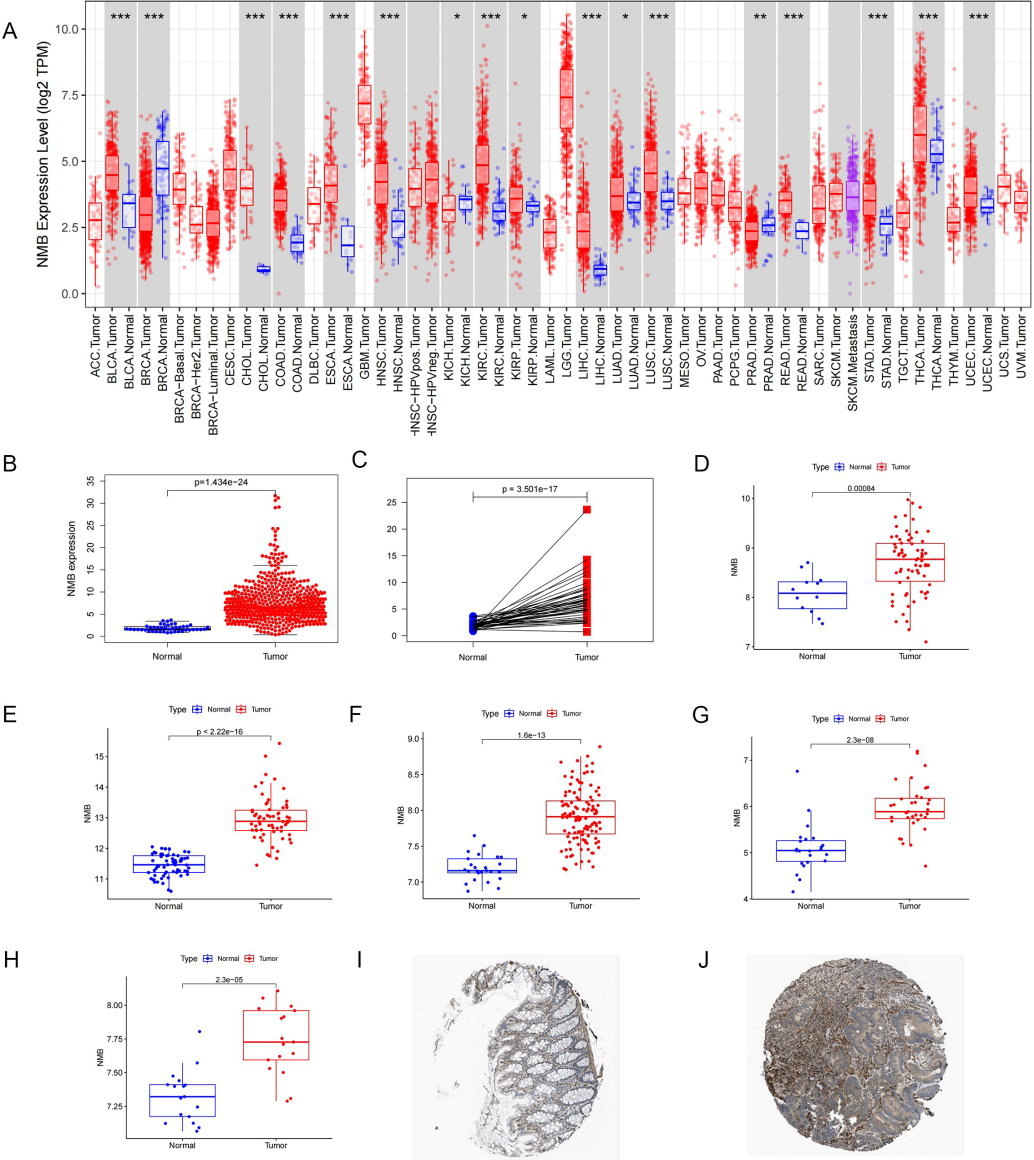
Analysis of NMB expression in database. Expression of NMB in pan-cancer from TCGA database **(A)**. The relative expression level of NMB was found in 44 normal cases and 568 CRC tissues in the TCGA database **(B)**. NMB expression was elevated in CRC tumor tissues compared to paired para-cancerous tissues **(C)**. Difference Analysis of NMB expression based on 8 data sets in GEO Database, including GSE9348**(D)**, GSE20482 **(E)**, GSE21510 **(F)**, GSE23878 **(G)**, GSE32323 **(H)**. Immunohistochemical analysis showed that the expression of NMB in CRC tissues was higher than that in normal colorectal tissues in HPA database **(I, J)** *P<0.05; **P<0.01; ***P<0.001.

Comprehensive analysis of NMB expression patterns in CRC was performed using multiple public databases. Initial examination of TCGA data revealed significantly elevated NMB mRNA levels in tumor tissues compared to both normal controls (p < 0.001, **Figures 1A, B**) and paired para-cancerous tissues (p < 0.001, **Figure 1C**). These findings were further corroborated by independent GEO datasets, which consistently demonstrated NMB overexpression across multiple CRC cohorts (p < 0.001, **Figures 1D-H**). Protein level validation using the Human Protein Atlas (HPA) database revealed that the expression of NMB was significantly increased in CRC specimens compared with healthy colorectal tissues (**Figures 1I, J**), collectively establishing NMB as a significantly upregulated marker in CRC.

**Figure 2:**
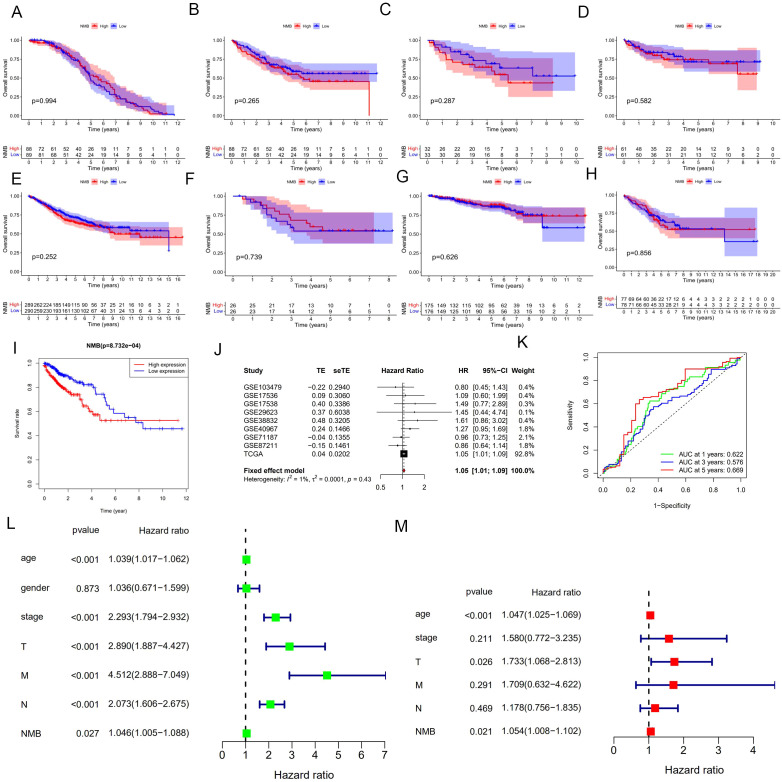
Correlation analysis of NMB expression with prognosis and clinical features in the database. Survival analysis based on eight datasets from the GEO database, including GSE17536 **(A)**, GSE17538 **(B)**, GSE29623 **(C)**, GSE38832 **(D)**, GSE40967 **(E)**, GSE71187 **(F)**, GSE87211 **(G)**, GSE103479 **(H)**, and TCGA database**(I)**. Meta-analysis of prognosis in patients with CRC **(J)**. ROC curve for NMB expression, AUC of NMB at 1, 3, 5 years were 0.622, 0.576 and 0.669 **(K)**. Univariate and multivariate cox regression analysis in the TCGA **(L, M)**. *P<0.05; **P<0.01; ***P<0.001.

To assess the prognostic significance of NMB, survival analyses were conducted using Kaplan-Meier methodology. TCGA dataset analysis revealed a significant association between elevated NMB expression and reduced overall survival (p < 0.001, **Figure 2I**), a trend consistently observed across GEO datasets (**Figures 2A-H**). Meta-analysis of these results demonstrated a hazard ratio (HR) of 1.0459 (95% CI: 1.0067-1.0865, z = 2.31, p = 0.0211), with forest plot visualization confirming NMB as a high-risk prognostic marker in CRC (**Figure 2J**). The overall meta-analysis estimate yielded an HR of 1.05 (95% CI: 1.01-1.09), supporting NMB’s role in CRC progression.

Diagnostic evaluation of NMB was performed through ROC curve analysis, incorporating expression data from 44 normal and 568 tumor samples. The AUC values demonstrated NMB’s diagnostic potential, with AUC = 0.622 at 1 year, 0.576 at 3 years, and 0.669 at 5 years (**Figure 2K**), indicating its substantial diagnostic value in CRC detection and monitoring.

### NMB is an independent predictor of poor survival in CRC

Following rigorous quality control measures, which involved the exclusion of cases with incomplete or ambiguous clinical data, a cohort of 472 colorectal cancer (CRC) patients was subjected to comprehensive survival analysis. Initial univariate Cox regression analysis identified several significant prognostic indicators (**Figure 2L**), including: advanced age (HR = 1.039, 95% CI: 1.017-1.062, p < 0.001), advanced tumor stage (HR = 2.293, 95% CI: 1.794-2.932, p < 0.001), higher T classification (HR = 2.890, 95% CI: 1.887-4.427, p < 0.001), lymph node metastasis (HR = 2.073, 95% CI: 1.606-2.675, p < 0.001), distant metastasis (HR = 4.512, 95% CI: 2.888-7.049, p < 0.001), and elevated NMB expression (HR = 1.046, 95% CI: 1.005-1.088, p = 0.027).

Multivariate Cox regression analysis, adjusting for potential confounding factors, revealed three independent predictors of reduced overall survival (OS) (**Figure 2M**): age (HR = 1.470, 95% CI: 1.025-2.113, p < 0.001), T classification (HR = 1.733, 95% CI: 1.068-2.813, p = 0.026), and high NMB expression (HR = 1.054, 95% CI: 1.008-1.102, p = 0.021). These results demonstrate that NMB expression maintains its prognostic significance even when controlling for established clinical parameters, suggesting its potential utility as a complementary biomarker in CRC risk stratification.

### Upregulation of NMB was associated with poor prognosis

**Figure 3:**
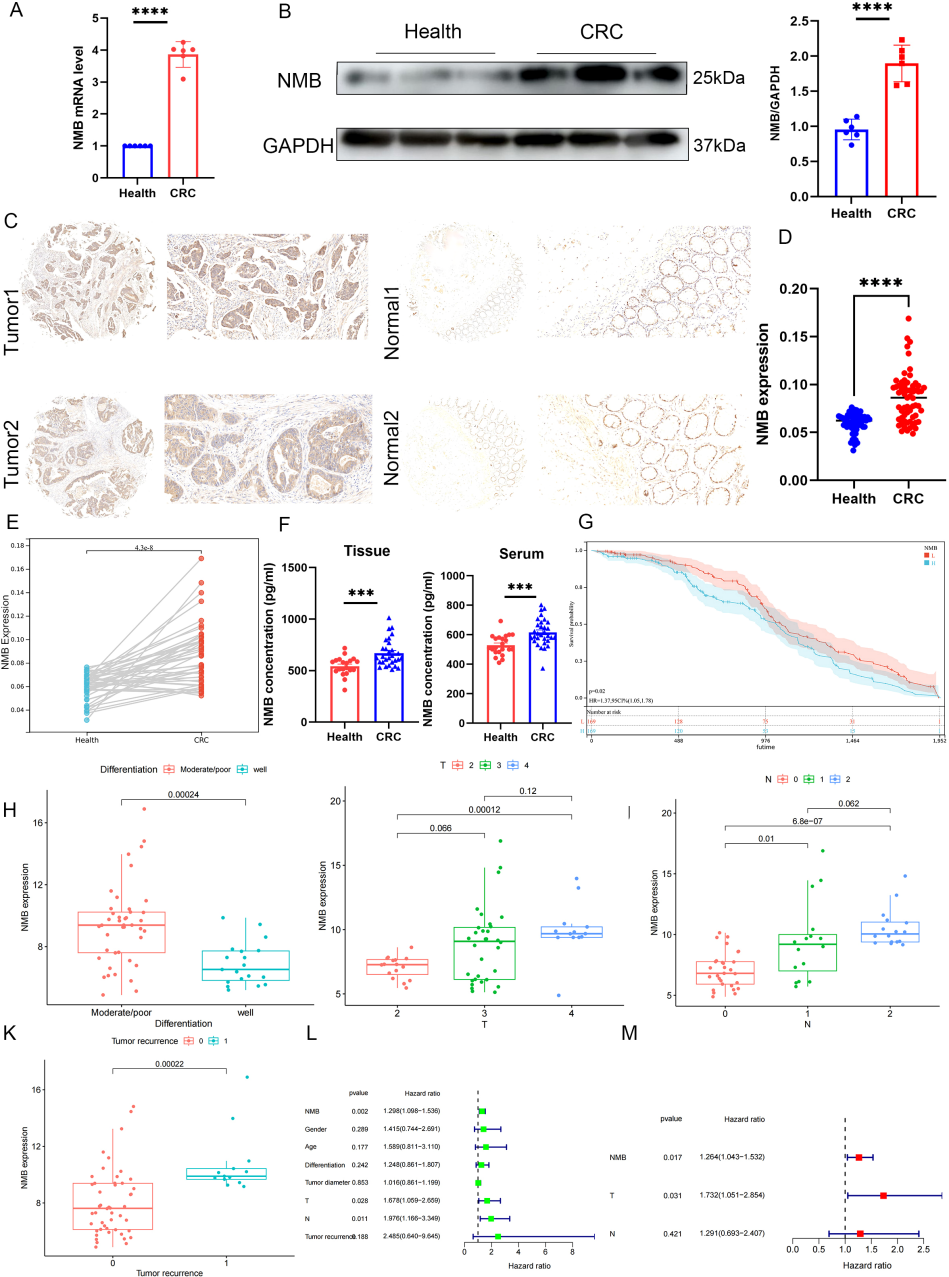
NMB expression is elevated and suggests a poor prognosis in CRC patients. The differential expression of NMB in CRC and normal colorectal tissues was analyzed by qRT-PCR and western bolt (n=6, p<0.001) **(A, B)**. Immunohistochemical analysis of NMB in tissue **(C-E)**. The expression of NMB in tissue homogenates and serum samples was detected by ELISA **(F)**. KM curve analysis of 61 CRC patients **(G)**. Correlation analysis of clinical features **(H-K)**. Univariate and multifactorial COX analysis of OS in CRC patients **(L-M)**.

To elucidate the functional significance of NMB in CRC, we employed a multi-modal experimental approach. qRT-PCR analysis demonstrated significant upregulation of NMB mRNA in tumor tissues relative to matched paracancerous controls (**Figure 3A**). Protein-level validation through Western blotting revealed markedly elevated NMB expression in CRC specimens compared to normal colorectal mucosa (**Figure 3B**).

Immunohistochemical examination of tissue microarrays from 61 CRC cases showed predominant cytoplasmic localization of NMB protein, with significantly higher positive staining rates in tumor tissues versus normal controls (p < 0.001). Comparative analysis between CRC tissues and adjacent non-tumor tissues confirmed this overexpression pattern (p < 0.001) (**Figures 3C-E**). ELISA quantification of tissue homogenates (p < 0.001) and serum samples (p < 0.001) further substantiated elevated NMB levels in CRC patients (**Figure 3F**).

Survival analysis using the Kaplan-Meier method demonstrated that high NMB expression correlated with significantly reduced overall survival (**Figure 3G**). Correlation studies identified significant associations between NMB expression levels and key clinicopathological parameters, including tumor differentiation, T stage, N stage, and recurrence status (**Figures 3H-K**). However, no significant correlations were observed with patient age, gender, or tumor diameter (**Supplementary Figures 1A-C**). Both univariate and multivariate Cox regression analyses established NMB expression as an independent prognostic indicator for overall survival in CRC (**Figures 3L, M**).

### Construction of the prognostic signature for CRC patients

**Figure 4:**
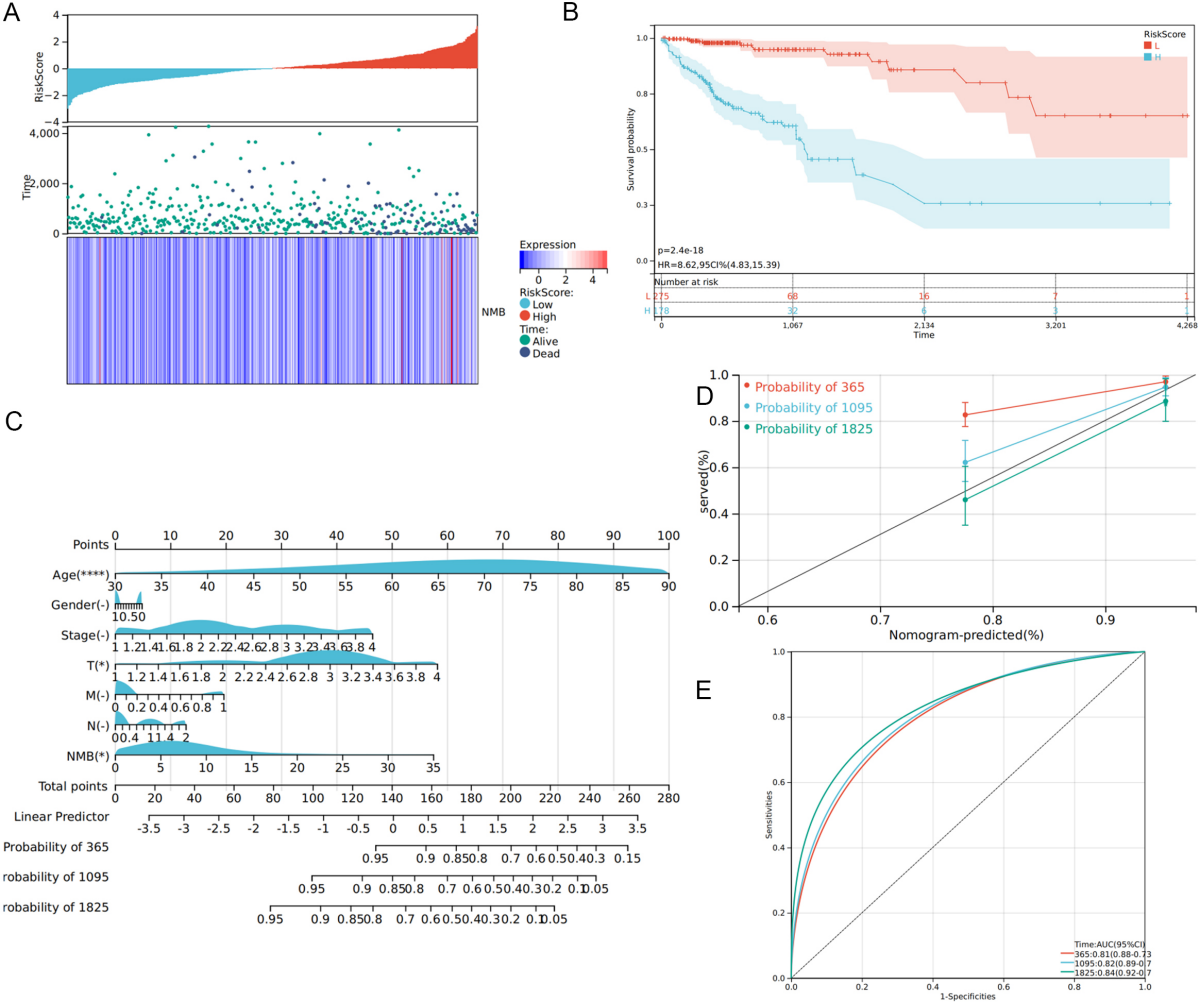
Construction of prognostic model. Distribution of risk scores of high- and low-risk CRC patients **(A)**. The K-M curve reflects that the OS of high-risk CRC patients is significantly lower than that of low-risk patients (P <0.001) **(B)**. Calculate the scores of each item of CRC patients according to the nomogram, and the total scores obtained after addition can predict the 1-, 3- and 5-year survival probability **(C)**. The 1-, 3- and 5-year calibration curve of the nomogram **(D)**. The ROC curve of 1-, 3- and 5-year nomogram (AUC = 0.81 of 1 year, AUC = 0.82 of 3 years, AUC = 0.84 of 5 years) **(E)**.

Utilizing Cox regression analysis, we constructed a prognostic model for CRC incorporating NMB expression levels and established clinical parameters, including age, gender, TNM classification, and tumor stage. The model demonstrated robust predictive performance, with a concordance index (C-index) of 0.79 (95% confidence interval: 0.73-0.84, p < 0.001). Risk stratification analysis revealed a clear distribution of patient risk scores, categorized from low to high risk (**Figure 4A**). Subsequent survival analysis using the Kaplan-Meier method confirmed significantly reduced overall survival in the high-risk group compared to low-risk patients (**Figure 4B**).

To enhance clinical applicability, we developed a comprehensive nomogram based on the prognostic model (**Figure 4C**). This graphical tool enables clinicians to calculate individual patient scores and estimate survival probabilities at 1-, 2-, and 3-year intervals, thereby supporting personalized treatment decisions. The nomogram’s predictive accuracy was validated through calibration curves, which demonstrated excellent agreement between predicted and observed survival rates at 1, 2, and 3 years (**Figure 4D**). ROC curve analysis further substantiated the model’s strong predictive capacity, with AUC values of 0.81, 0.82, and 0.84 for 1-, 2-, and 3-year survival predictions, respectively (**Figure 4E**).

### Knocking out NMB inhibit the progression of CRC

To elucidate the functional significance of NMB in CRC, we systematically evaluated its expression and biological effects *in vitro*. qRT-PCR and Western blot analyses were performed to assess endogenous NMB levels across normal colonic mucosal cells and a panel of CRC cell lines. The results demonstrated variable expression patterns, with HCT116 cells exhibiting the highest NMB levels, followed by moderate expression in SW480 cells and minimal expression in HT29, CaCo2, and SW620 lines (**Supplementary Figures 2A, B**).

**Figure 5:**
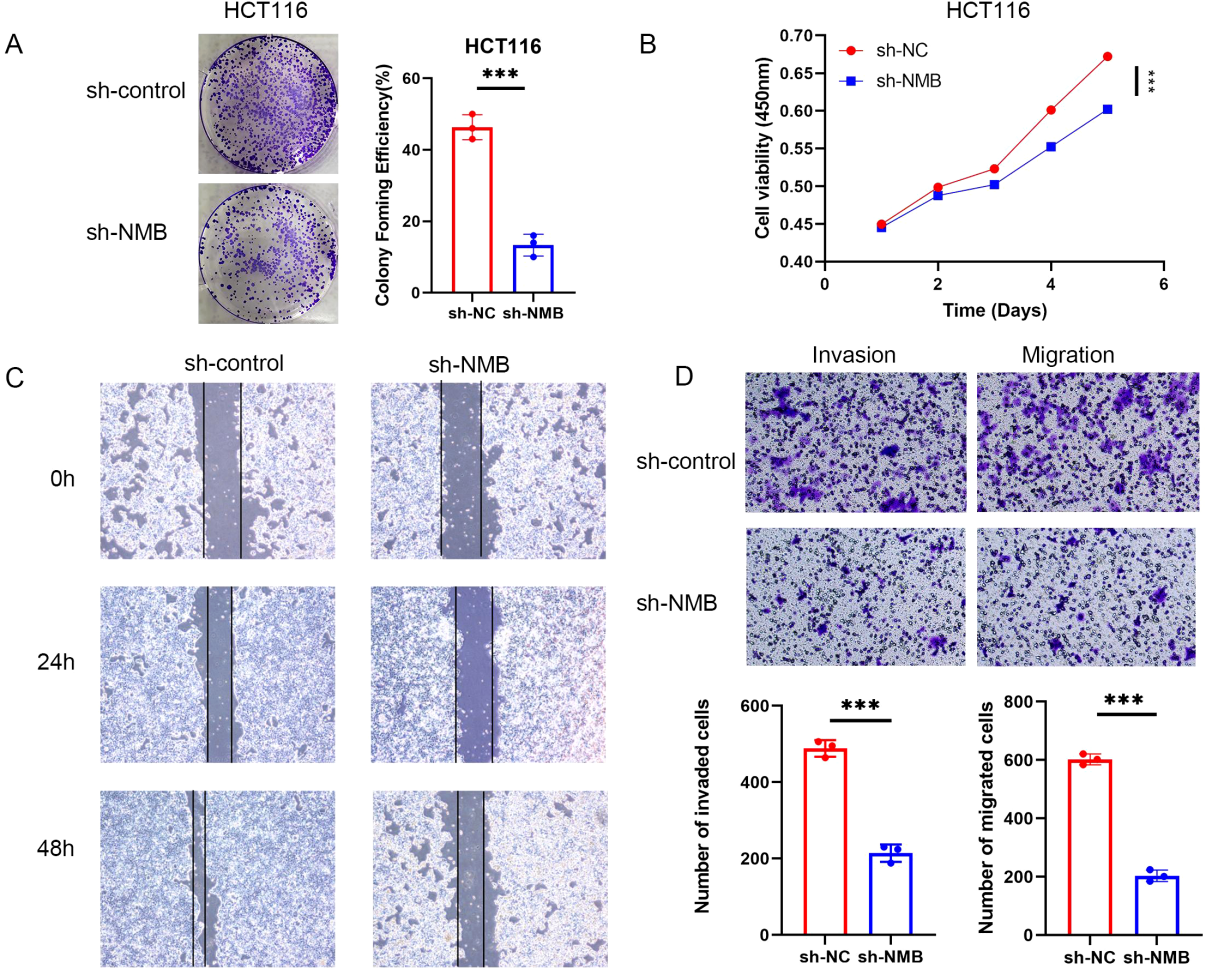
Verification of the role of NMB in the regulation of CRC *in vitro*. NMB regulates the proliferation, migration and invasion of CRC cells. Cloning experiment analysis **(A)**. CCK-8 assay analysis **(B)**. Experimental analysis of wound-healing assay **(C)**. Experimental analysis of migration and invasion **(D)**. *P<0.05; **P<0.01; ***P<0.001.

For functional characterization, we established stable NMB knockdown in HCT116 cells using specific shRNA constructs, with shRNA-NC serving as the negative control. Western blot validation performed 48 hours post-transfection confirmed efficient NMB suppression (**Supplementary Figure 2C**). Functional assays revealed that NMB depletion significantly impaired cellular proliferation, as evidenced by reduced colony formation capacity (**Figure 5A**) and decreased metabolic activity in MTT assays (**Figure 5B**).

Further investigations demonstrated that NMB knockdown markedly attenuated the migratory potential of HCT116 cells, as quantified through both wound healing assays and Transwell migration tests. Additionally, NMB suppression significantly reduced cellular invasiveness, as measured by Transwell invasion assays (**Figure 5D**). These findings collectively indicate that NMB plays a crucial role in promoting CRC cell proliferation, migration, and invasion.

### Relationship between NMB expression and immune infiltration

**Figure 6:**
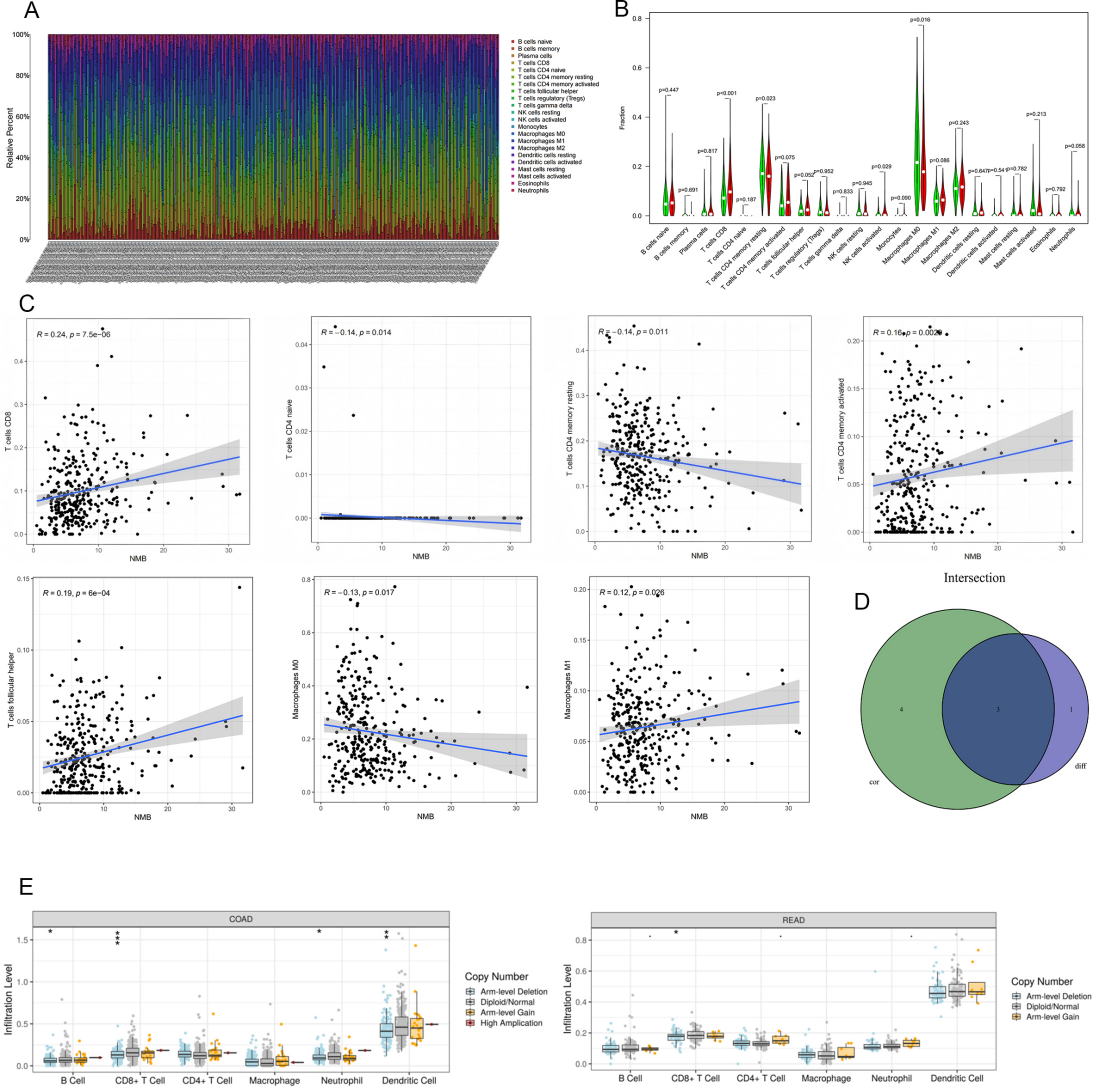
Relationship Between NMB Expression and Immune Infiltration in CRC. The number of immune cells in each sample **(A)**. Results of differential analysis of immune cells. Green represents the low expression group and red represents the high expression group **(B)**. NMB was significantly correlated with T cells CD8(R=0.24,p<0.001),T cells CD4 naive(R=-0.14,p=0.014),T cells CD4 memory resting(R=-0.14,p=0.011),T cells CD4 memory activated(R=0.16,p=0.0029),T cells follicular helper(R=0.19,p<0.001),Macrophages M0(R=-0.13,p=0.017) and Macrophages M1(R=0.12,p=0.026) **(C)**;The intersection of the two test results **(D)**. The relationships between infltration levels of 6 immune cells and copy number of NMB in CRC **(E)**.*P<0.05; **P<0.01; ***P<0.001.

The tumor microenvironment (TME) has emerged as a critical determinant of clinical outcomes across various malignancies, including breast cancer (11), ovarian cancer, CRC, and gastric cancer. To explore the immunological implications of NMB in CRC, we conducted comprehensive analyses of its association with immune cell infiltration. Initial quantification of immune cell populations revealed distinct profiles across patient samples (**Figure 6A**).

Comparative analysis between NMB expression levels and immune cell infiltration demonstrated significant differences in several immune cell subtypes. Specifically, CD8^+^ T cells (p < 0.001), resting memory CD4^+^ T cells (p = 0.023), activated NK cells (p = 0.029), and M0 macrophages (p = 0.016) showed differential infiltration patterns between high and low NMB expression groups (**Figure 6B**). Correlation analysis further identified significant associations between NMB expression and multiple immune cell populations, including positive correlations with CD8^+^ T cells (R = 0.24, p < 0.001), activated memory CD4^+^ T cells (R = 0.16, p = 0.0029), and follicular helper T cells (R = 0.19, p < 0.001), as well as negative correlations with naive CD4^+^ T cells (R = -0.14, p = 0.014), resting memory CD4^+^ T cells (R = -0.14, p = 0.011), and M0 macrophages (R = -0.13, p = 0.017) (**Figure 6C**).

Integration of these analyses revealed three key immune cell types consistently associated with NMB expression: CD8^+^ T cells, resting memory CD4^+^ T cells, and M0 macrophages (**Figure 6D**). Furthermore, investigation of NMB mutational variants demonstrated their influence on the infiltration patterns of six major leukocyte populations, including B cells, CD4^+^ T cells, CD8^+^ T cells, macrophages, neutrophils, and dendritic cells (**Figure 6E**). These findings collectively indicate that NMB plays a significant role in modulating immune infiltration within the CRC tumor microenvironment.

### Correlation between expression level of NMB and immune marker sets

To elucidate the immunological role of NMB in CRC, we conducted a comprehensive analysis of its association with immune cell infiltration using the TIMER database. The study encompassed a broad spectrum of immune markers, including those for T cells, CD8+ T cells, B cells, monocytes, neutrophils, NK cells, tumor-associated macrophages (TAMs), M1/M2 macrophages, and dendritic cells. Additionally, we examined functional T cell subsets, specifically Th1, Th2, Treg, Tfh, Th17, and exhausted T cells.

Our analysis revealed significant associations between NMB expression levels and immune marker sets across multiple immune cell types (**Supplementary Table 1**). Following adjustment for tumor purity, we identified distinct immune marker profiles correlated with NMB expression in colon and rectal cancers. In colon cancer, the following markers demonstrated strong correlations (P < 0.001; correlation coefficient ≥ 0.40): CD3E, HLA-DPB1, CD3D, CD79A, TGFB1, CD2, HLA-DPA1, ITGAX, HLA-DRA, CD86, CCR7, CD19, NRP1, HAVCR2, CTLA4, and TBX21. Similarly, in rectal cancer, significant correlations were observed with CD3E, HAVCR2, IL10, CD1C, CEACAM8, CCR7, STAT3, CTLA4, ITGAM, HLA-DPB1, FOXP3, CCL2, KIR3DL1, and STAT1 (P < 0.001; Cor value ≥ 0.40).

These findings demonstrate that NMB expression exhibits distinct patterns of association with immune markers in CRC, suggesting its potential role in modulating the tumor immune microenvironment in CRC subtypes.

### Deubiquitin enzyme USP21 affects the ubiquitin level of NMB

Emerging evidence has established that ubiquitin-specific proteases (USPs) serve as critical regulators of immune cell function and response within the TME (12, 13). Specifically, ubiquitin-specific protease 21 (USP21), a prominent member of the USP family, has been implicated in modulating TME dynamics in CRC (14). Given the established association between NMB and CRC TME, we hypothesized a potential regulatory relationship between NMB and USP21.

**Figure 7:**
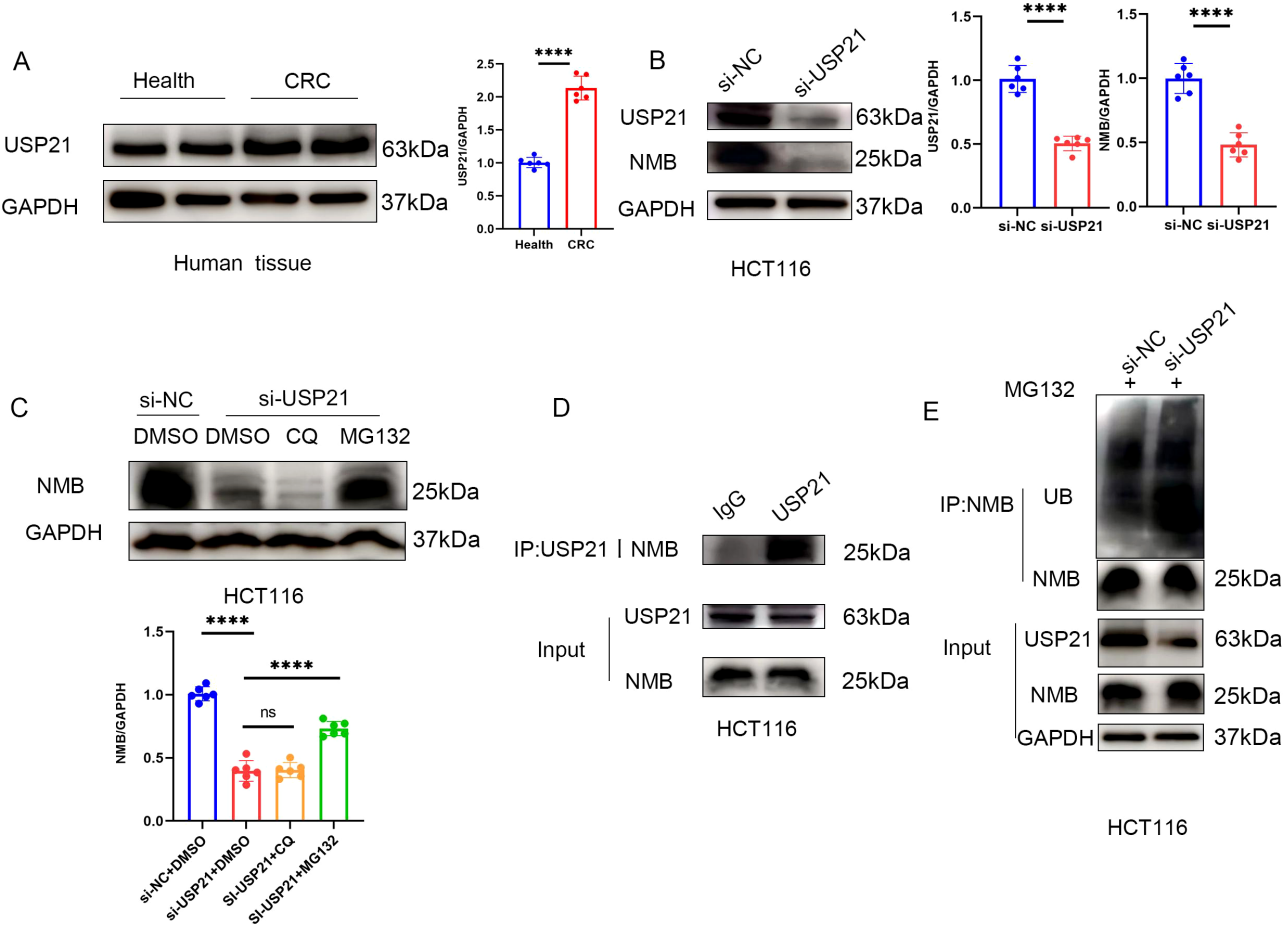
USP21 regulates the ubiquitin level of NMB. Western blot suggests that the expression in USP21 CRC tissue is higher than that in healthy colorectal tissue (n=6, p<0.001) **(A)**. The expression of NMB decreased after knocking down USP21 in HCT116 cells (n=6, p<0.001) **(B)**, In HCT116 cells, MG132 could reverse the decrease of NMB expression induced by knocking down USP21, but CQ had no effect **(C)**. Co-IP suggested that USP21 could bind to NMB **(D)**. Knocking down USP21 can increase the level of ubiquitin in NMB **(E)**.

Experimental validation demonstrated significantly elevated USP21 expression in CRC tissues (**Figure 7A**). Notably, USP21 silencing resulted in a marked reduction in NMB protein levels (**Figure 7B**). To elucidate the underlying mechanism of NMB regulation, we employed pharmacological inhibitors targeting distinct protein degradation pathways. Treatment with the proteasome inhibitor MG132 effectively rescued NMB downregulation induced by USP21 knockdown, whereas the autophagy inhibitor chloroquine (CQ) exhibited no significant effect (**Figure 7C**).

Further mechanistic investigations revealed a direct physical interaction between NMB and USP21, as confirmed by co-immunoprecipitation assays (**Figure 7D**). Importantly, USP21 depletion led to increased ubiquitination of NMB (**Figure 7E**). These findings collectively demonstrate that elevated NMB expression in CRC is mechanistically linked to USP21-mediated deubiquitination.

### NMB affects CRC by regulating NF- κ B signaling pathway

**Figure 8:**
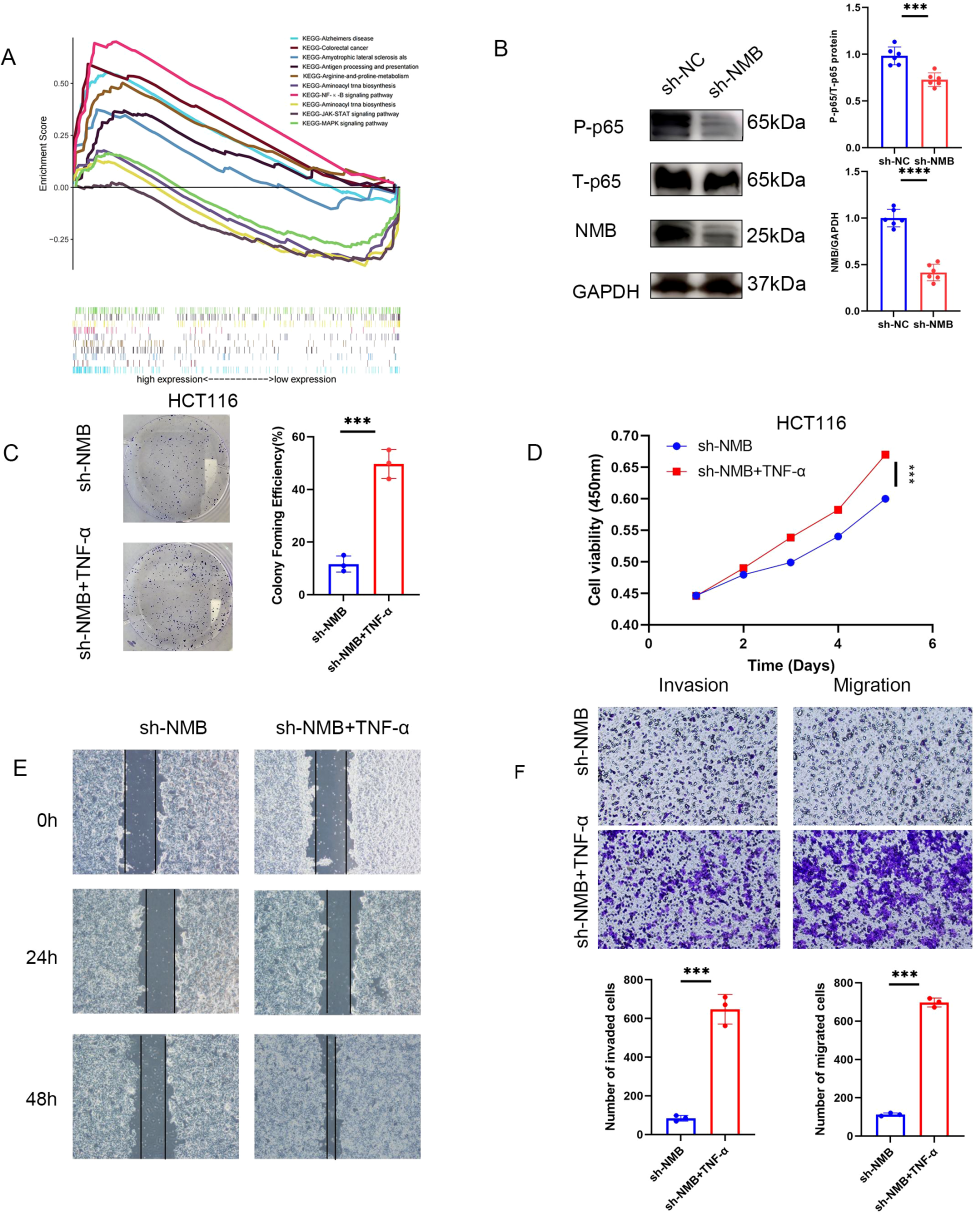
NMB affects the development of CRC by regulating NF-κB signal pathway. A merged enrichment plot from GSEA including enrichment score and gene sets **(A)**. Western blot suggested that knocking down NMB could significantly reduce the phosphorylation of p65 at S536 site, and the total p65 decreased slightly **(B)**. In the cell function test, the antitumor effect induced by knocking down NMB could be reversed by adding TNF (NF-K B activator). Including cloning experiment analysis **(C)**, CCK-8 assay analysis **(D)**, experimental analysis of wound-healing assay **(E)**, experimental analysis of migration and invasion **(F)**. *P<0.05; **P<0.01; ***P<0.001.

To elucidate the molecular mechanisms underlying NMB-mediated regulation of CRC, we performed GSEA comparing transcriptional profiles between low and high NMB expression groups. The analysis demonstrated significant enrichment of the NF-κ B signaling pathway in high NMB phenotypes, with CRC-related pathways also showing marked enrichment, further supporting the functional relevance of NMB in CRC pathogenesis (**Figure 8A**).

To investigate whether NMB exerts its oncogenic effects through NF-κ B pathway modulation, we examined the phosphorylation status of p65 at serine 536 (S536). NMB knockdown resulted in a substantial reduction in p65 phosphorylation, indicating impaired NF-κ B activation (**Figure 8B**). Functional validation experiments utilizing TNF-α, a potent NF-κ B pathway activator, demonstrated that pathway stimulation could effectively rescue the suppression of tumor cell proliferation, migration, and invasion induced by NMB knockdown (**Figures 8C-F**). In addition, given the established prognostic significance of RAS and RAF proto-oncogenes in CRC (15), we found that NMB suppression markedly reduced RAS and RAF protein expression levels, suggesting a potential regulatory role in the RAS/RAF-MAPK signaling axis (**Supplementary Figure 3**).

### Analysis of drug sensitivity

**Figure 9:**
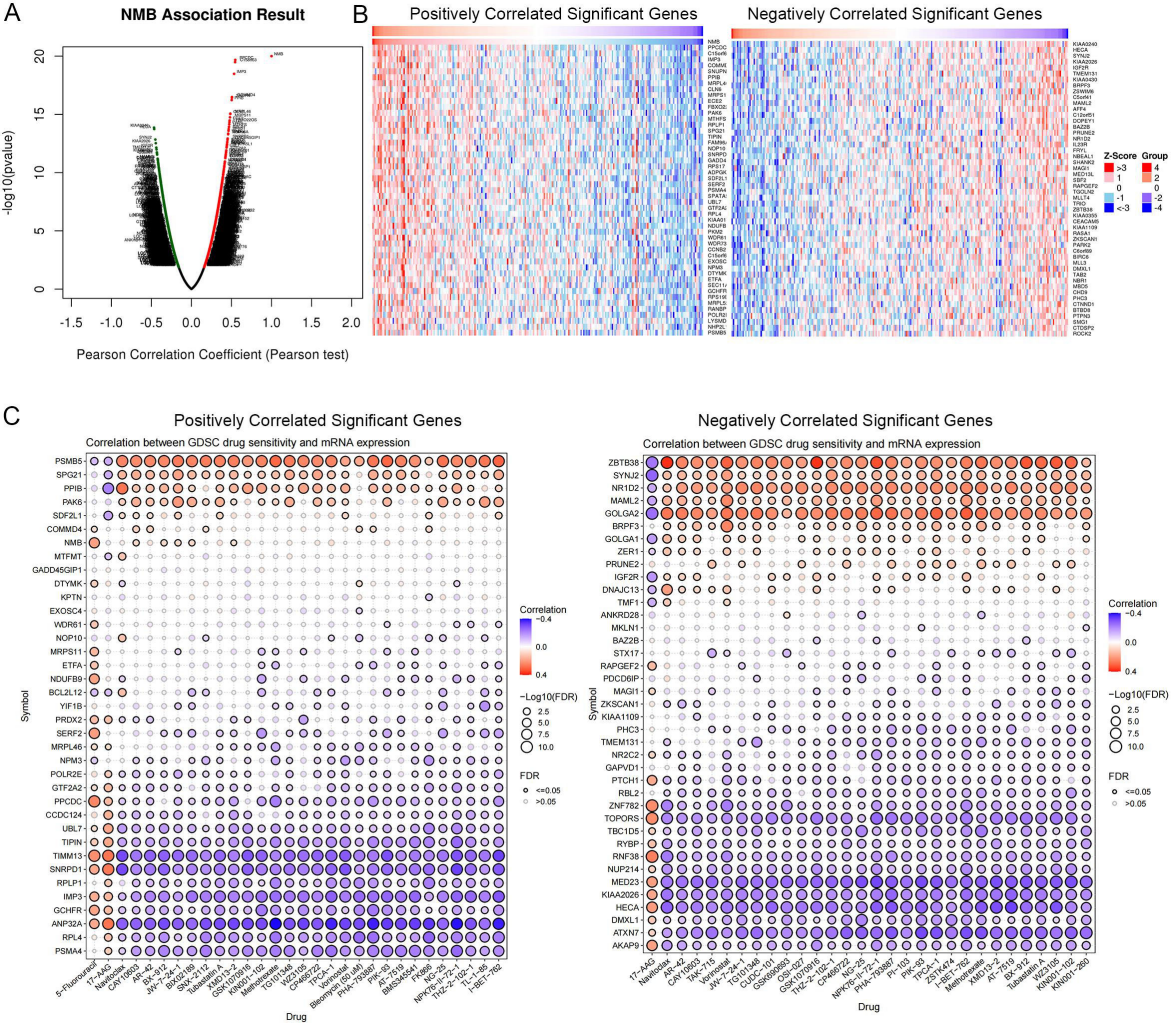
Drug sensitivity analysis. NMB co-expression genes in CRC cohort were identified by Linked Omics **(A)**. Heatmaps showing top 50 genes positively and negatively correlated with NMB in CRC. Red dot, positively correlated gene; blue dot, negatively correlated genes **(B)**. Correlation between NMB co-expression gene set and drug resistance, red indicates a positive correlation, and higher gene expression can lead to an increase in drug resistance, while blue indicates the opposite. **(C)**.

Using a co-expression network analysis, we identified the top 100 genes most closely associated with NMB expression (**Figures 9A, B**). Pearson correlation analysis was then employed to assess the relationship between mRNA expression levels of these genes and drug sensitivity (IC50 values). The results revealed that genes positively correlated with NMB expression exhibited a significant association with 5-fluorouracil (5-FU) resistance. Furthermore, NMB co-expressed genes demonstrated notable correlations with resistance to multiple chemotherapeutic agents and targeted therapies, including methotrexate, tubastatin, and vorinostat (**Figure 9C**).

## Discussion

Despite a notable decline in cancer mortality rates since 1991 (5), malignancies continue to represent a substantial global health burden, accounting for significant morbidity and mortality worldwide (1). CRC, the most common neoplasm of the digestive system, is predominantly diagnosed through colonoscopy (16), although this diagnostic approach presents several limitations (17). Early detection remains paramount for enhancing survival outcomes, mitigating disease progression, and preventing tumor recurrence. The implementation of precision medicine, as emphasized by Christopher E. Barbieri, has revolutionized patient care through the identification of predictive biomarkers and therapeutic targets (18). Recent investigations by Fang-Ze Wei and Wei Xu have identified CLCA1 and SNHG11 as promising prognostic markers and therapeutic targets in CRC, underscoring the critical role of biomarkers in disease management (19–21). Building upon these findings, Matusiak et al. reported elevated NMB expression in CRC tissues (10). In the present study, we have further elucidated the functional role of NMB in CRC pathogenesis, investigated the molecular mechanisms underlying its overexpression, and explored the potential regulatory mechanisms through which NMB modulates CRC progression.

We retrieved transcriptomic profiles and corresponding clinical survival data for 711 CRC patients from TCGA database, comprising 612 cases (44 normal and 568 tumor samples). Utilizing the survival, Beeswarm, and Limma R packages, we generated scatter plots and paired comparative analyses, which demonstrated significantly elevated NMB expression levels in CRC tissues compared to normal controls. To validate these findings, we analyzed multiple independent datasets from the GEO database (GSE9348, GSE20482, GSE21510, GSE23878, GSE32323), consistently confirming the upregulation of NMB in CRC. Through Kaplan-Meier survival analysis, we established a significant correlation between elevated NMB expression and reduced OS in CRC patients. ROC curve analysis further substantiated the prognostic significance of NMB expression. Multivariate Cox proportional hazards regression analysis identified NMB as an independent predictor of poor OS in CRC. These findings suggest that differential NMB expression not only provides novel insights into CRC pathogenesis but also represents a promising diagnostic and prognostic biomarker for CRC management.

To further elucidate the functional significance of NMB in CRC, we conducted comprehensive molecular analyses using clinical samples. Tissue microarray analysis of paired tumor and normal tissues from 61 CRC patients revealed significantly elevated NMB expression through immunohistochemical staining. Quantitative assessment using qRT-PCR demonstrated increased NMB RNA levels in tumor tissues, while ELISA quantification detected higher NMB concentrations in patient serum samples. Western blot analysis further confirmed the upregulation of NMB protein expression in CRC tissues. Kaplan-Meier survival analysis established a significant correlation between elevated NMB expression and unfavorable clinical outcomes in CRC patients. Correlation studies with clinicopathological parameters demonstrated that NMB expression levels were significantly associated with tumor differentiation grade, T and N classification, and recurrence status. Multivariate Cox proportional hazards regression analysis identified NMB expression as an independent prognostic factor for OS in CRC patients.

To improve clinical utility and enhance prognostic accuracy in CRC management, we developed a comprehensive prognostic model capable of calculating individualized risk scores for each patient. This model demonstrated significant predictive value for OS, with high-risk patients exhibiting markedly worse clinical outcomes compared to their low-risk counterparts. Furthermore, we established a nomogram incorporating key prognostic variables to estimate survival probabilities at 1-, 3-, and 5-year intervals. The predictive accuracy of this nomogram was validated through calibration curves and ROC analysis, confirming its robust performance in clinical outcome prediction. Furthermore, we evaluated the role of NMB in CRC *in vitro*. We downregulated NMB expression in HCT116 cells. Subsequent colony formation and MTT assay demonstrated that reducing NMB inhibited CRC cell proliferation, while abnormal NMB expression enhanced proliferation. Migration and invasion assay further confirmed that NMB expression regulates the migration and invasion capabilities of CRC cells. To sum up, our clinical data analysis and related experiments show that the increased expression of NMB in CRC effectively promotes tumor proliferation, migration and invasion.

Emerging evidence underscores the pivotal role of TME in tumorigenesis, progression, and patient prognosis (22–24). To elucidate the immunomodulatory functions of NMB in CRC, we systematically analyzed its associations with immune infiltration and immune-related biomarkers. Through comprehensive analyses, we identified significant correlations between NMB expression and specific immune cell populations, including CD8^+^ T cells, resting memory CD4^+^ T cells, and M0 macrophages. Further intersection of these results provided robust evidence of NMB’s involvement in immune regulation. Utilizing the TIMER database, we investigated the relationship between NMB expression and immune marker sets across CRC subtypes. In colon cancer, significant associations (P < 0.001; correlation coefficient ≥ 0.40) were established with markers such as CD3E, HLA-DPB1, CD3D, CD79A, TGFB1, CD2, HLA-DPA1, ITGAX, HLA-DRA, CD86, CCR7, CD19, NRP1, HAVCR2, CTLA4, and TBX21. Similarly, in rectal cancer, NMB expression exhibited significant correlations (P < 0.001; correlation coefficient ≥ 0.40) with markers including CD3E, HAVCR2, IL10, CD1C, CEACAM8, CCR7, STAT3, CTLA4, ITGAM, HLA-DPB1, FOXP3, CCL2, KIR3DL1, and STAT1. These findings suggest potential mechanisms by which NMB may modulate immune cell function within the CRC microenvironment.

Emerging evidence highlights the critical role of ubiquitin-specific proteases (USPs), the largest and most functionally diverse class of deubiquitinating enzymes (DUBs), in modulating immune cell activity and TME dynamics (25–27). USP21, a member of the USP family, is located on chromosome 1q21. It is characterized by its C-terminal catalytic DUB domain. Fang Jun and others have shown that USP21 plays an important role in TME, treatment response and clinical prognosis of CRC (14). Our study shows that NMB plays an important role in regulating immune cell infiltration in TME of CRC. In this way, we question whether the increase of NMB in CRC is closely related to USP21. First, by detecting the protein level of USP21 in healthy people and CRC patients, we found that USP21 increased in CRC, which was consistent with Fang Jun’s research. Subsequently, NMB decreased significantly after silencing USP21 in HCT116 cell line. As a classical deubiquitin enzyme (28), the effect of USP21 on NMB is largely related to the ubiquitin proteasome pathway. Proteasome inhibitor MG132 could significantly reverse the down regulation of NMB induced by USP21 knockdown, while autophagy inhibitor CQ had no effect. Co-IP further proved that NMB and USP21 can combine with each other. Ubiquitin level detection showed that silencing USP21 in CRC cells significantly enhanced the ubiquitin level of NMB. These data suggest that the increased expression of NMB in CRC is closely related to the enhanced deubitization ability of USP21.

To elucidate the molecular mechanisms underlying NMB’s role in CRC, we conducted KEGG pathway analysis using GSEA4.1.0 software. The results revealed significant enrichment of the NF-κB signaling pathway in high-NMB expression phenotypes, along with CRC-related pathways, suggesting a strong association between NMB and CRC pathogenesis. Furthermore, NMB demonstrated involvement in multiple KEGG pathways, including JAK-STAT signaling (29, 30), MAPK signaling pathway (31, 32), Alzheimers disease, Amyotrophic lateral sclerosis als, Antigen processing and presentation, Arginine-and-proline-metabolism, Aminoacyl trna biosynthesis, Aminoacyl trna biosynthesis. To validate these findings, we investigated NMB’s regulatory effects in CRC cell lines. NMB knockdown markedly reduced phosphorylation of p65 at Ser536 without significantly altering total p65 levels, indicating that NMB modulates NF-κ B activation through phosphorylation regulation. Functional assays demonstrated that the NF-κ B activator TNF-α effectively counteracted the inhibitory effects of NMB silencing on tumor cell proliferation, migration, and invasion. These findings collectively suggest that NMB promotes CRC progression through NF-κ B pathway regulation. In addition, the critical prognostic role of RAS and RAF proto-oncogenes in CRC pathogenesis prompted our investigation into NMB’s regulatory function. Experimental knockdown of NMB in CRC models yielded a marked reduction in both RAS and RAF protein expression. This functional relationship positions NMB as a potential co-biomarker for RAS/RAF-mutated CRC cases, while simultaneously revealing novel opportunities to overcome MAPK inhibitor resistance through NMB-targeted intervention strategies.

Extensive research has established a strong correlation between aberrant gene expression and chemotherapy resistance (33, 34). Despite significant advancements in cancer treatment modalities, 5-fluorouracil (5-FU) remains a cornerstone in colorectal cancer (CRC) therapy and a key component of combination chemotherapy regimens. However, the clinical application of oral 5-FU monotherapy has been discontinued due to its unpredictable gastrointestinal absorption and suboptimal pharmacokinetic profile. Consequently, optimizing 5-FU-based combination therapies has emerged as a critical focus in cancer research. Current clinical practice often combines 5-FU with oxaliplatin and/or irinotecan to enhance therapeutic efficacy, albeit with increased toxicity profiles (35). To explore NMB’s potential role in chemotherapy resistance, we employed the Linked Omics Platform to identify NMB co-expressed genes. We selected the top 50 genes positively and negatively correlated with NMB expression for drug sensitivity analysis. Notably, the gene set positively associated with NMB expression demonstrated a significant correlation with 5-FU resistance, providing novel insights for improving 5-FU combination therapy. Furthermore, our analysis revealed NMB’s association with resistance mechanisms and targeted therapy responses to multiple chemotherapeutic agents, including methotrexate (36), tubastatin A (37), vorinostat (38) and so on.

In conclusion, our comprehensive database analysis and experimental findings collectively establish NMB as a novel biomarker for colorectal cancer (CRC). This study not only elucidates the regulatory mechanisms underlying NMB’s role in CRC progression but also provides a foundation for developing innovative systemic treatment strategies.

However, several limitations should be noted in this investigation. First, the relatively limited sample size requires validation through larger, more diverse patient cohorts to strengthen the reliability of our conclusions. Second, although we have demonstrated that USP21 regulates NMB expression via deubiquitination in CRC, the potential contributions of other ubiquitin-specific proteases (USPs) remain to be investigated. Future studies should employ advanced techniques, such as mass spectrometry-based proteomics, to systematically examine the broader USP family. Third, the specific molecular pathways of NMB-induced drug resistance could not be further analyzed in this study, this represents a critical focus for our ongoing research. future work will combine transcriptomic/proteomic analyses of resistant cell lines with animal models to identify key pathways and develop strategies for improving CRC treatment efficacy.

## Conclusion

In conclusion, our integrated analysis of public databases and clinical CRC cases has identified NMB as a significant prognostic biomarker for colorectal cancer. Through *in vitro* studies, we have demonstrated NMB’s regulatory role in CRC pathogenesis. To enhance clinical utility, we developed a highly predictive nomogram for patient survival assessment. Mechanistically, we revealed that USP21-mediated deubiquitination upregulates NMB expression in CRC, which subsequently modulates p65 phosphorylation to activate the NF-κ B signaling pathway, thereby influencing CRC progression.

Our chemotherapeutic response analysis established a notable correlation between NMB expression patterns and 5-FU resistance, highlighting its potential impact on therapeutic efficacy and its relevance in optimizing 5-FU-based combination therapies. This bioinformatics-driven research, complemented by experimental validation, provides critical insights for improving CRC diagnosis and developing targeted therapeutic strategies. The bioinformatics component of this study has been made publicly available as a preprint on Research Square, facilitating scientific discourse and collaboration (39).

## Data Availability

The original contributions presented in the study are included in the article/**Supplementary Material**. Further inquiries can be directed to the corresponding authors.

## Supplementary material

Supplementary Figure 1 Correlation analysis of clinical features. *P<0.05; **P<0.01; ***P<0.001.

Supplementary Figure 2 Construction of CRC cell line with NMB stable knockdown. **(A, B)** The expression of NMB in CRC cell lines was analyzed by qRT-PCR and Western blot. **(C)** Western blot to verify the inefficiency of NMB knocking. * p < 0.05; ** p < 0.01; *** p < 0.001.

Supplementary Figure 3 NMB Modulates RAS and RAF Expression in CRC Cells. *P<0.05; **P<0.01; ***P<0.001.
