# Supplementary figures and images for: NMB promotes the progression of colorectal cancer by regulating the NF-κB/P65 signaling pathway
Source: Front Immunol. 2025 May 23;16:1596451. doi: 10.3389/fimmu.2025.1596451 (PMC12141226; doi:10.3389/fimmu.2025.1596451)

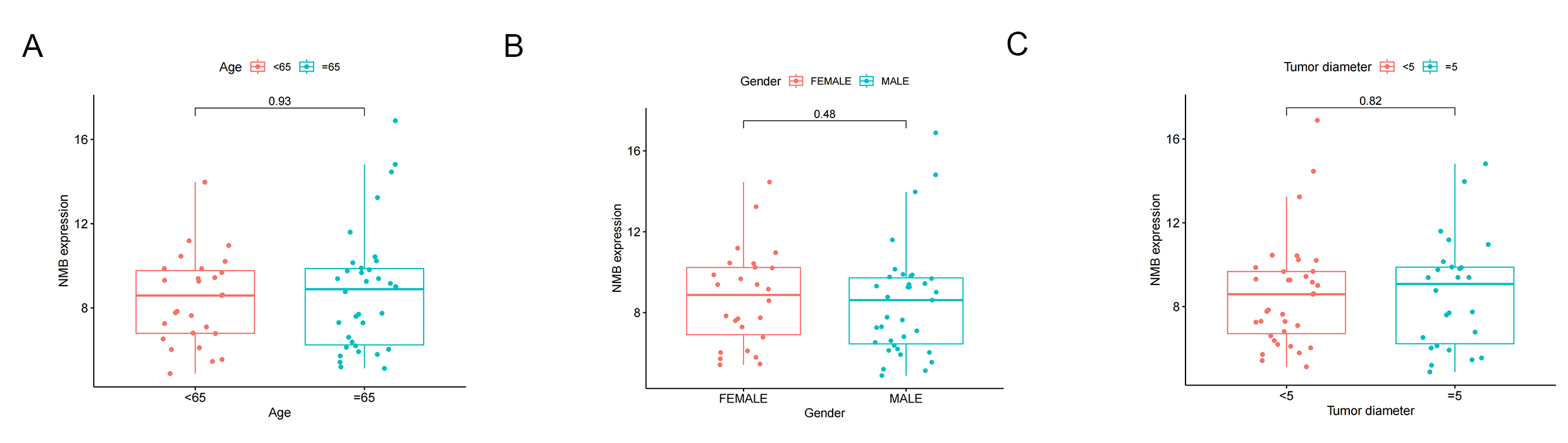

Supplement: Supplementary Figure 1 — Correlation analysis of clinical features. *P<0.05; **P<0.01; ***P<0.001. [file Image1.jpeg]

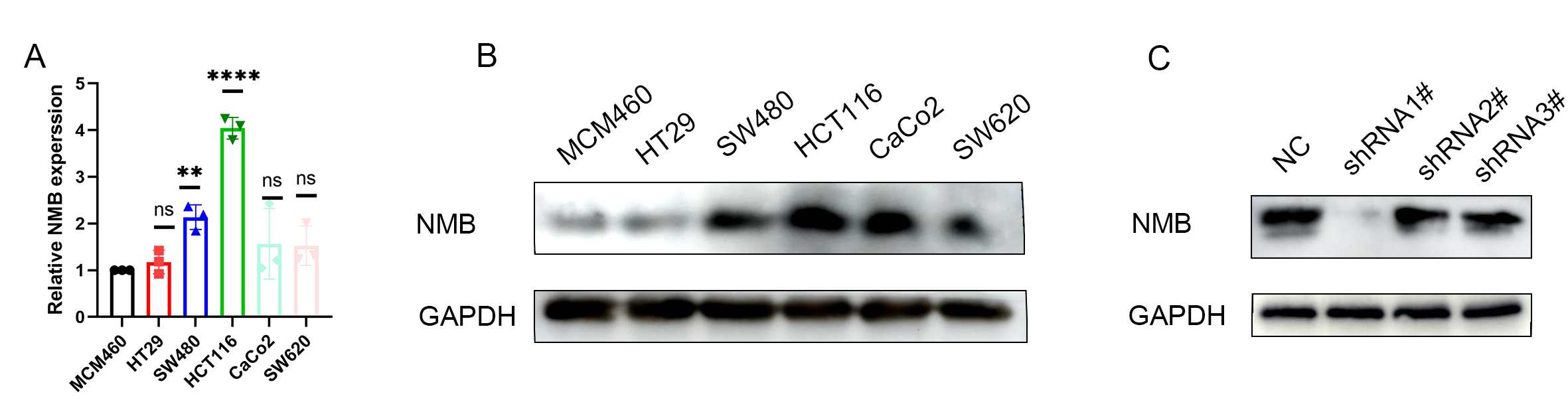

Supplement: Supplementary Figure 2 — Construction of CRC cell line with NMB stable knockdown. (A, B) The expression of NMB in CRC cell lines was analyzed by qRT-PCR and Western blot. (C) Western blot to verify the inefficiency of NMB knocking. * p < 0.05; ** p < 0.01; *** p < 0.001. [file Image2.jpeg]

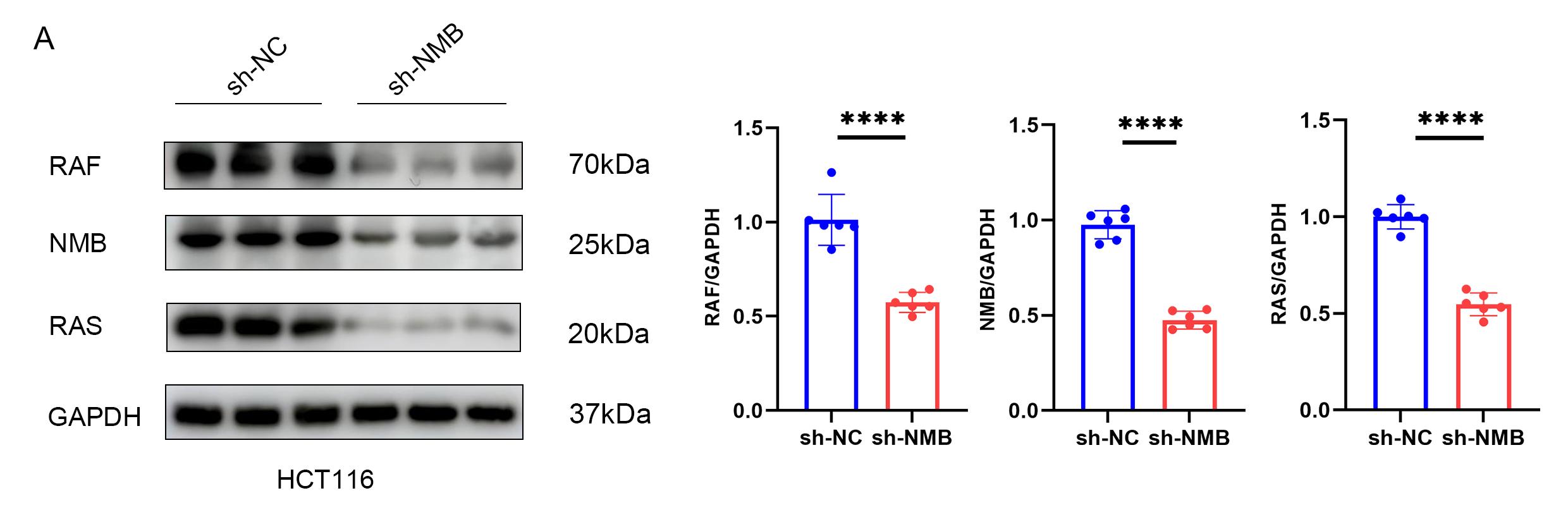

Supplement: Supplementary Figure 3 — NMB Modulates RAS and RAF Expression in CRC Cells. *P<0.05; **P<0.01; ***P<0.001. [file Image3.jpeg]
