# Supplementary material for: NMB promotes the progression of colorectal cancer by regulating the NF-κB/P65 signaling pathway
Source: Front Immunol. 2025 May 23;16:1596451. doi: 10.3389/fimmu.2025.1596451 (PMC12141226; doi:10.3389/fimmu.2025.1596451)
Supplement: Supplementary file 4 [file Table1.docx]

Supplementary Table1│Correlation analysis between NMB and relate genes and markers of immune cells in TIMER.

| **Description** | **Gene markers** | **COAD** | | | | | | **READ** | | | | | |
| --- | --- | --- | --- | --- | --- | --- | --- | --- | --- | --- | --- | --- | --- |
|  |  | **None** | | **Purity** | | **Age** | | **None** | | **Purity** | | **Age** | |
|  |  | **Cor** | **P** | **Cor** | **P** | **Cor** | **P** | **Cor** | **P** | **Cor** | **P** | **Cor** | **P** |
| CD8 + Tcell | CD8A | 0.119 | 0.011 | -0.397 | 0.000 | -0.093 | 0.047 | -0.093 | 0.233 | -0.402 | 0.000 | 0.024 | 0.761 |
|  | CD8B | 0.15 | 0.001 | -0.221 | 0.000 | -0.015 | 0.757 | -0.035 | 0.657 | -0.127 | 0.134 | -0.041 | 0.598 |
| T cell (general) | CD3D | 0.062 | 0.187 | -0.447 | 0.000 | 0.057 | 0.224 | -0.082 | 0.295 | -0.412 | 0.000 | 0.045 | 0.560 |
|  | CD3E | 0.043 | 0.354 | -0.477 | 0.000 | 0.051 | 0.277 | -0.126 | 0.105 | -0.444 | 0.000 | 0.104 | 0.183 |
|  | CD2 | 0.083 | 0.077 | -0.435 | 0.000 | 0.061 | 0.191 | -0.093 | 0.234 | -0.404 | 0.000 | 0.05 | 0.521 |
| B cell | CD19 | 0.025 | 0.594 | -0.414 | 0.000 | -0.052 | 0.271 | -0.063 | 0.422 | -0.35 | 0.000 | -0.056 | 0.471 |
|  | CD79A | 0.005 | 0.912 | -0.443 | 0.000 | -0.025 | 0.596 | -0.198 | 0.011 | -0.443 | 0.000 | 0.108 | 0.165 |
| Monocyte | CD86 | 0.077 | 0.099 | -0.42 | 0.000 | 0.056 | 0.220 | -0.046 | 0.556 | -0.407 | 0.000 | -0.058 | 0.456 |
|  | CSF1R | 0.017 | 0.713 | -0.366 | 0.000 | 0.078 | 0.099 | -0.124 | 0.113 | -0.414 | 0.000 | 0.011 | 0.888 |
| TAM | CCL2 | 0.062 | 0.182 | -0.354 | 0.000 | 0.058 | 0.214 | -0.051 | 0.510 | -0.474 | 0.000 | -0.01 | 0.903 |
|  | CD68 | 0.072 | 0.123 | -0.317 | 0.000 | 0.063 | 0.181 | -0.055 | 0.478 | -0.355 | 0.000 | 0.026 | 0.735 |
|  | IL10 | 0.093 | 0.046 | -0.303 | 0.000 | 0.106 | 0.024 | 0.024 | 0.762 | -0.381 | 0.000 | -0.026 | 0.743 |
| M1 Macrophage | NOS2 | 0.176 | 0.000 | -0.131 | 0.008 | 0.076 | 0.105 | -0.003 | 0.967 | -0.064 | 0.451 | 0.112 | 0.151 |
|  | IRF5 | 0.091 | 0.051 | -0.093 | 0.061 | -0.009 | 0.852 | 0.114 | 0.145 | -0.117 | 0.167 | -0.002 | 0.979 |
|  | PTGS2 | 0.125 | 0.007 | -0.221 | 0.000 | 0.062 | 0.190 | 0.109 | 0.162 | -0.258 | 0.002 | -0.077 | 0.322 |
| M2 Macrophage | CD163 | 0.051 | 0.279 | -0.389 | 0.000 | 0.106 | 0.024 | 0.081 | 0.296 | -0.379 | 0.000 | -0.015 | 0.845 |
|  | VSIG4 | 0.085 | 0.070 | -0.355 | 0.000 | 0.109 | 0.020 | 0.010 | 0.894 | -0.327 | 0.000 | 0.052 | 0.502 |
|  | MS4A4A | 0.074 | 0.111 | -0.379 | 0.000 | 0.109 | 0.020 | -0.078 | 0.320 | -0.407 | 0.000 | 0.013 | 0.866 |
| Neutrophils | CEACAM8 | 0.099 | 0.034 | 0.103 | 0.038 | 0.076 | 0.106 | 0.152 | 0.051 | 0.109 | 0.202 | 0.116 | 0.137 |
|  | ITGAM | -0.021 | 0.655 | -0.355 | 0.000 | 0.054 | 0.253 | -0.096 | 0.218 | -0.361 | 0.000 | -0.017 | 0.828 |
|  | CCR7 | 0.028 | 0.552 | -0.415 | 0.000 | -0.052 | 0.267 | -0.090 | 0.246 | -0.375 | 0.000 | -0.102 | 0.192 |
| Natural killer cell | KIR2DL1 | 0.121 | 0.009 | -0.189 | 0.000 | 0.053 | 0.262 | -0.038 | 0.627 | -0.174 | 0.039 | 0.077 | 0.321 |
|  | KIR2DL3 | 0.091 | 0.053 | -0.196 | 0.000 | 0.127 | 0.007 | -0.083 | 0.288 | -0.186 | 0.028 | -0.087 | 0.267 |
|  | KIR2DL4 | 0.118 | 0.012 | -0.322 | 0.000 | 0.112 | 0.017 | -0.04 | 0.609 | -0.348 | 0.000 | 0.039 | 0.617 |
|  | KIR3DL1 | 0.113 | 0.015 | -0.249 | 0.000 | 0.083 | 0.079 | 0.007 | 0.930 | 0.161 | 0.057 | -0.067 | 0.391 |
|  | KIR3DL2 | -0.003 | 0.954 | -0.264 | 0.000 | 0.056 | 0.236 | -0.133 | 0.088 | -0.295 | 0.000 | 0.091 | 0.245 |
|  | KIR3DL3 | 0.039 | 0.405 | -0.063 | 0.203 | 0.016 | 0.732 | -0.077 | 0.327 | -0.084 | 0.323 | -0.063 | 0.422 |
|  | KIR2DS4 | 0.108 | 0.021 | -0.116 | 0.019 | 0.048 | 0.307 | 0.093 | 0.243 | -0.098 | 0.250 | 0.116 | 0.138 |
| Dendritic cell | HLA-DPB1 | 0.062 | 0.188 | -0.450 | 0.000 | 0.115 | 0.014 | -0.120 | 0.123 | -0.414 | 0.000 | 0.010 | 0.896 |
|  | HLA-DQB1 | 0.154 | 0.000 | -0.334 | 0.000 | 0.056 | 0.041 | 0.057 | 0.468 | -0.267 | 0.001 | 0.033 | 0.676 |
|  | HLA-DRA | 0.102 | 0.030 | -0.422 | 0.000 | 0.103 | 0.028 | -0.049 | 0.530 | -0.445 | 0.000 | -0.003 | 0.972 |
|  | HLA-DPA1 | 0.108 | 0.021 | -0.431 | 0.000 | 0.118 | 0.012 | -0.121 | 0.120 | -0.451 | 0.000 | -0.006 | 0.937 |
|  | CD1C | 0.012 | 0.805 | -0.331 | 0.000 | -0.068 | 0.151 | -0.218 | 0.005 | -0.394 | 0.000 | 0.000 | 0.997 |
| Th1 | NRP1 | 0.010 | 0.827 | -0.414 | 0.000 | -0.016 | 0.728 | -0.069 | 0.377 | -0.382 | 0.000 | -0.141 | 0.070 |
|  | ITGAX | 0.023 | 0.628 | -0.427 | 0.000 | 0.016 | 0.732 | -0.017 | 0.830 | -0.410 | 0.000 | -0.022 | 0.779 |
|  | TBX21 | 0.084 | 0.074 | -0.400 | 0.000 | 0.102 | 0.030 | -0.098 | 0.209 | -0.358 | 0.000 | 0.067 | 0.389 |
|  | STAT1 | 0.088 | 0.059 | -0.274 | 0.000 | 0.076 | 0.108 | 0.009 | 0.911 | -0.318 | 0.000 | -0.068 | 0.385 |
|  | IFNG | 0.144 | 0.002 | -0.241 | 0.000 | 0.151 | 0.001 | -0..096 | 0.219 | -0.307 | 0.000 | 0.075 | 0.339 |
|  | TNF | 0.093 | 0.046 | -0.255 | 0.000 | 0.063 | 0.180 | 0.171 | 0.028 | -0.313 | 0.000 | -0.082 | 0.295 |
| Th2 | GATA3 | 0.027 | 0.567 | -0.364 | 0.000 | -0.024 | 0.614 | -0.061 | 0.432 | -0.331 | 0.000 | -0.017 | 0.828 |
|  | STAT6 | -0.139 | 0.003 | 0.012 | 0.815 | 0.011 | 0.818 | -0.130 | 0.096 | 0.060 | 0.482 | 0.174 | 0.025 |
|  | STAT5A | 0.044 | 0.345 | -0.161 | 0.001 | 0.037 | 0.434 | 0.052 | 0.503 | -0.222 | 0.008 | 0.042 | 0.595 |
|  | IL13 | 0.140 | 0.003 | -0.197 | 0.000 | 0.007 | 0.886 | 0.024 | 0.763 | -0.219 | 0.009 | -0.035 | 0.655 |
| Tfh | BCL6 | 0.005 | 0.916 | -0.357 | 0.000 | 0.011 | 0.807 | -0.131 | 0.093 | -0.222 | 0.008 | 0.062 | 0.427 |
|  | IL21 | 0.054 | 0.246 | -0.134 | 0.007 | 0.083 | 0.078 | 0.016 | 0.841 | -0.195 | 0.021 | -0.066 | 0.399 |
| Th17 | STAT3 | -0.054 | 0.250 | -0.225 | 0.000 | -0.062 | 0.186 | -0.131 | 0.093 | -0.245 | 0.004 | -0.090 | 0.250 |
|  | IL17A | 0.017 | 0.711 | -0.012 | 0.814 | 0.023 | 0.630 | -0.036 | 0.650 | 0.061 | 0.471 | 0.158 | 0.042 |
| Treg | FOXP3 | 0.052 | 0.265 | -0.382 | 0.000 | 0.018 | 0.707 | -0.117 | 0.133 | -0.383 | 0.000 | 0.038 | 0.626 |
|  | CCR8 | 0.017 | 0.721 | -0.336 | 0.000 | -0.021 | 0.657 | -0.088 | 0.258 | -0.390 | 0.000 | -0.100 | 0.201 |
|  | STAT5B | -0.077 | 0.100 | -0.054 | 0.279 | -0.121 | 0.010 | -0.073 | 0.648 | -0.203 | 0.016 | -0.117 | 0.132 |
|  | TGFB1 | 0.138 | 0.003 | -0.435 | 0.000 | 0.043 | 0.357 | 0.076 | 0.331 | -0.393 | 0.000 | -0.001 | 0.989 |
